# The Anticonvulsant Effect of Nonsteroidal Anti‐Inflammatory Drug, Fenoprofen, in Pentylenetetrazole‐Induced Epileptic Rats: Behavioral, Histological, and Biochemical Evidence

**DOI:** 10.1002/prp2.70072

**Published:** 2025-02-05

**Authors:** Maryam Rahimi‐Tesiye, Hassan Rajabi‐Maham, Vahid Azizi, Abdolkarim Hosseini

**Affiliations:** ^1^ Department of Animal Sciences and Marine Biology Faculty of Life Sciences and Biotechnology Shahid Beheshti University Tehran Iran

**Keywords:** antioxidant activity, antioxidative stress, endogenous antioxidant, Nissl staining, Nrf2/HO‐1 signaling, oxidative damage

## Abstract

This study aimed to evaluate the anticonvulsant properties of fenoprofen on the experimental model of pentylenetetrazole (PTZ)‐induced epilepsy. Male Wistar rats were randomly grouped into five, and the kindling model was induced by intraperitoneal injection of PTZ 35 (mg/kg) every other day for 1 month. Aside from the control and PTZ groups, three groups received intraperitoneal injections of fenoprofen at doses of 10, 20, and 40 (mg/kg) before each PTZ injection. Rats were challenged with PTZ 70 (mg/kg) 1 week after kindling development. Then rats were subjected to deep anesthesia, and serum and brain samples were prepared. Oxidative stress (OS) markers (malondialdehyde, superoxide dismutase, and glutathione peroxidase) were measured in serum samples. Hippocampal tissue was used to investigate the relative expression of OS‐related genes (*nuclear factor* [*erythroid‐derived 2*]‐*like 2* (*Nrf2*)/*heme oxygenase 1* (*Hmox1*)) *and* histological studies. Seizure behavior was assessed based on Lüttjohann's score. In treated groups, the number of myoclonic jerks and generalized tonic–clonic seizure (GTCS) duration decreased significantly, while myoclonic jerks and GTCS latency increased compared with the PTZ group. The biochemical evaluation revealed the antioxidative effects of fenoprofen. The decreased expression of *Nrf2*/*HO‐1* genes in the PTZ group was reversed after fenoprofen administration. The results of the histological study obtained from Nissl staining in the hippocampal tissue also confirmed the protective effect of fenoprofen. The anticonvulsant effects of fenoprofen seem to be through inhibition of OS‐related markers, induction of protective effect in hippocampal tissue, and activation of the Nrf2/HO‐1 signaling pathway.

## Graphical abstract

As shown in the figure, rats were subjected to fenoprofen (Fen) and PTZ injections for 1 month to induce the kindling model of epilepsy. After the PTZ challenge, their convulsive behaviors were recorded, and 24 h later, serum and brain samples were obtained. The relative expression of *Nrf2* and *HO‐1* genes as well as histological investigations were performed in hippocampus tissue sections using RT‐qPCR and Nissl staining. Oxidative stress markers were also measured in rat serum samples. As shown in the figure, after entering the laboratory and going through the adaptation period, rats were grouped and injected with saline, DMSO, PTZ, or different doses of fenoprofen along with PTZ every other day for a month to induce the kindling model of epilepsy. One week after the induction of the model, the rats were challenged with PTZ 70 (mg/kg), and their convulsive behaviors were recorded. After this stage, the brain and serum samples were prepared under deep anesthesia for further studies.

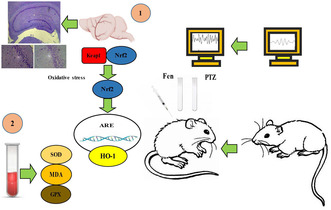

## 1 Introduction

Epilepsy is a life‐threatening neurological disorder affecting millions worldwide, and it is one of the most common disorders that cause a decrease in life expectancy and disability. Many people experience seizures during their lifetime. However, a high percentage of people have recurrent seizures, a condition known as epilepsy. Although various antiepileptic drugs (AEDs) have been introduced to treat or control epileptic attacks, the side effects of their long‐term use, such as dose‐dependent neurocognitive effects and idiosyncratic reactions, are discussed [1, 2].

Various factors stimulate the brain circuits for convulsive activities by changing the biophysical and chemical conditions of the brain microenvironment, including redox imbalance, cytokine expression, altered neurogenesis, gamma‐aminobutyric acid (GABA)/glutamate receptors imbalance, neuroinflammation, increase in reactive oxygen species (ROS), and ultimately oxidative stress (OS) [3, 4]. Excessive ROS elevation has been identified as a critical activator of the redox‐regulated proinflammatory transcription factors. These factors trigger inflammatory reactions by activating downstream signaling pathways. This is one of the mechanisms through which OS and inflammation interact and are involved in hyperexcitability [5, 6]. Therefore, targeting pathways related to OS or neuroinflammation can improve our knowledge in treating or identifying cellular and molecular pathways involved in epilepsy.

The Nuclear factor (erythroid‐derived 2)‐like 2 (Nrf2)/Heme oxygenase 1 (HO‐1), signaling pathway plays a significant role in response to inflammatory reactions. Nrf2 is sequestered in the cytoplasm by Kelch‐like ECH‐associated protein 1 (KEAP1) and regulates the expression of various genes and antioxidant factors under pathophysiological conditions. Activation of Nrf2 and its related factors is a critical antioxidant and anti‐inflammatory pathway that causes neuroprotection [7]. Once activated, Nrf2 separates from KEAP1 and is displaced to the nucleus, which induces the *HO*‐*1* gene expression. The Nrf2/HO‐1 signaling pathway has potent antioxidant and detoxification effects, thus inducing neuroprotection against OS and inflammatory reactions [8, 9].

Nonsteroidal anti‐inflammatory drugs (NSAIDs) with anti‐inflammatory, analgesic, and antipyretic effects mainly act on cyclooxygenases (COXs) to regulate the level of prostaglandins (PGs) or their receptors (PGRs). In this way, they can modulate neuronal hyperexcitability and subsequent neuronal firing [10]. Previous studies in different epilepsy models showed that pretreatment with NSAIDs such as Ibuprofen or Indomethacin had anticonvulsant effects. These drugs by reducing inflammation or OS‐related biomarkers, control the severity of seizures and cause partial recovery [11, 12]. As a propionic acid derivative and NSAID, Fenoprofen is used to treat rheumatoid arthritis, relieve pain, treat degenerative joint diseases, and reduce inflammation following surgery. Fenoprofen, a nonselective COX inhibitor, can limit PG synthesis and subsequently reduce the production of inflammatory mediators [13]. Other NSAIDs, such as Aspirin and Celecoxib, have also been shown to reduce seizure intensity and frequency as well as OS markers and, in some cases, have been reported to inhibit mossy fiber sprouting, neuronal death, and aberrant neurogenesis [10, 14].

Pentylenetetrazol (PTZ), as a GABA_A_ receptor antagonist, is used as a seizure‐inducing chemical in laboratory models. Repeated injection of a subconvulsant dose oF PTZ induces the kindling model in rodents, which is widely used in the study of epilepsy pathology and the effects of antiepileptic drugs [15]. PTZ induces oxidative and inflammatory damage in brain circuits and directly causes mitochondrial dysfunction, DNA damage, apoptosis, and induction of cognitive disorders [16]. As far as we know, there are no specific studies on the effect of fenoprofen on convulsive behaviors and OS‐related markers. In this study, we investigated the anticonvulsant and antioxidative effects of fenoprofen on the rat model of PTZ‐induced epilepsy.

Pentylenetetrazol (PTZ), as a GABA_A_ receptor antagonist, is used as a seizure‐inducing chemical in laboratory models. Repeated injection of a subconvulsant dose oF PTZ induces the kindling model in rodents, which is widely used in the study of epilepsy pathology and the effects of antiepileptic drugs [15]. PTZ induces oxidative and inflammatory damage in brain circuits and directly causes mitochondrial dysfunction, DNA damage, apoptosis, and induction of cognitive disorders [16]. As far as we know, there are no specific studies on the effect of fenoprofen on convulsive behaviors and OS‐related markers. In this study, we investigated the anticonvulsant and antioxidative effects of fenoprofen on the rat model of PTZ‐induced epilepsy.

## 2 Methods

### 2.1 Animals

Thirty male Wistar rats weighing 190 ± 20 g and aged 4 months were used in the present study. Rats were kept in a room with constant conditions of a 12‐h dark/light cycle, and 22°C ± 2°C. The rats were placed in standard polycarbonate cages, and food and water were provided throughout the experiment. All procedures followed the Guide for the Care and Use of Laboratory Animals published by the US National Institutes of Health. The study adhered to ARRIVE guidelines. It was also approved by the local ethics committee of Shahid Beheshti University (code no. IR.SBU.REC.1402.122).

### 2.2 Drugs

Fenoprofen calcium salt hydrate powder was provided by Sigma‐Aldrich Co., (USA) and was dissolved in Dimethyl sulfoxide (DMSO), prepared from Neutron Pharmachemical Co., (Iran). PTZ was supplied by Sigma‐Aldrich (USA) and was dissolved in normal saline.

### 2.3 Experimental Design and Behavioral Evaluation

**FIGURE 1:**
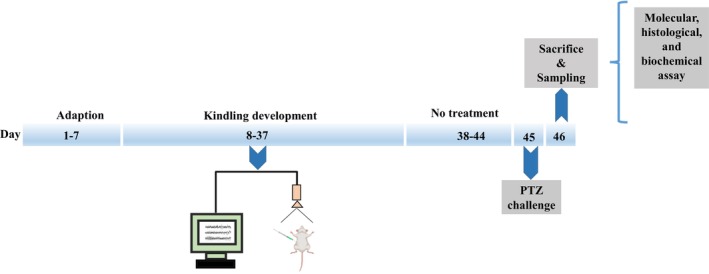
Schematic design of the test schedule includes creating a kindling model, drug injection, behavioral tests, and sampling.

Through the simple randomization approach, rats were divided into five groups of six as follows: (1) control group: received i.p injection of DMSO, followed by normal saline, after 30 min, (2) PTZ group: received i.p injection of DMSO followed by PTZ (35 mg/kg) after 30 min, (3–5) Fen groups: received i.p injections of fenprofen (10, 20, and 40 mg/kg) followed by PTZ (35 mg/kg) after 30 min. The selection of fenoprofen injection doses in this study was based on previous findings [17, 18]. All injections were done every other day for a month, and drugs were injected at a volume of 0.5 mL. After the second injection, rats were transferred into separate plastic cages, and their seizure behaviors were recorded for 1 h. Seizure behaviors were assessed using the Racine scale modified by Lüttjohann, Fabene, and van Luijtelaar [19], (1) head nodding, (2) wet‐dog shakes, (3) forelimb clonus, (4) forelimb clonus with rearing, (5) animals falling to the side, (6) jumping. One week after kindling development, rats were challenged with PTZ (70 mg/kg). The number and onset time of myoclonic jerks (NMJ and MJL) and the duration and delay of generalized tonic–clonic seizure onset (GTCS‐L and GTCS‐D) were measured (Figure 1) [18, 19]. It should be noted that the experimenter was not aware of which rat belonged to which experimental group, so this research was done in a double‐blind manner.

### 2.4 Sample Preparation

Twenty‐four hours after completing the experiment, the animals were subjected to deep anesthesia with a mixture of ketamine (90 mg/kg) and xylazine (10 mg/kg) (Alfasan Co., Netherlands), and following cardiac puncture, the thoracic blood was collected. Blood samples were clotted for half an hour at room temperature and then centrifuged at 3500 *g* for 15 min. Serums were collected and kept at −20°C for further analysis [20]. Then animals were quickly sacrificed and their brain samples were obtained. Part of the hippocampus was kept at −80°C for reverse transcription polymerase chain reaction (RT‐PCR), and the other part was preserved in 10% formalin for histological studies [21].

### 2.5 Estimation of OS Markers

To measure oxidative stress markers: malondialdehyde (MDA), superoxide dismutase (SOD), and glutathione peroxidase (GPX) in animal serum samples, conventional kits (Novin Navand Salamat Co., Iran) and a microplate reader (BioTek Epoch2‐USA) were used. MDA evaluation was performed according to Nalondi Kit (Novin Navand Salamat Co., Iran) instruction. MDA was measured using the TBARS (Thiobarbituric Acid Reactive Substances) method, which is a method for measuring lipid peroxidation. The procedure was briefly carried out in the following steps: (1) first a solution including the 200 mL sample (or standard) with 800 mL working solution was prepared. (2) The samples were placed in water at 95°C for 45 min. (3) Then the samples were kept on ice for 10 min to cool. (4) The samples were centrifuged at 3000 rpm for 15 min, and the supernatant was used to examine the reaction between thiobarbituric acid and MDA at a wavelength of 550 nm. The results are reported as nM/mL.

The Nasdox kit (Novin Navand Salamat Co., Iran) was used to measure SOD enzyme activity. The basis of this test is the inhibition of the autoxidation reaction of pyrogallol, a compound that is oxidized in the presence of oxygen. After preparing the solution according to the kit steps and mixing it with the samples, they were incubated for 5 min at room temperature, and then measured at a wavelength of 405 nm and reported as mU/mL.

Nagpix GPX activity assay kit (Novin Navand Salamat Co., Iran) was used to assess GPx levels. To evaluate GPx content, Nagpix GPX activity assay kit (Novin Navand Salamat Co., Iran) was used. After preparing the working and standard solutions (based on the kit instructions), the test was performed in 3 steps. (1) 40 μL of reagent 1 was added to 50 μL of the samples (and standard) and incubated for 15 min at room temperature. (2) 10 μL of reagent 2 was added to the samples (or standard) and the absorbance was evaluated at 340 nm. (3) After 5–10 min of incubation at room temperature, the absorbance was measured again at 340 nm. The final absorbance was reported as mU/mL.

### 2.6 Histological Studies

#### 2.6.1 Nissl Staining

The brains fixed in 10% formalin were subjected to Nissl staining according to the previous instruction [22]. Briefly, brain samples were dehydrated and embedded in paraffin, and then coronal sections (5 μm) were taken by microtome (Sakura‐Japan). Hippocampal sections were stained with cresyl violet in line with routine protocol to assess morphological changes and cell density. An Olympus Optical Co. LTD microscope (Japan) was used for digital imaging. For cell counting and image scaling, ImageJ software, version 1.5.3. was used.

### 2.7 Molecular Measurement

The brain samples were first homogenized using a homogenizer device (Tomy Co., USA). Total RNAs were extracted using the total RNA extraction kit (Parstous Co., Iran). Possible DNA contaminants were removed using DNase I treatment (Parstous Co., Iran). A Nanodrop device (BioTek Epoch2‐USA) was used to determine the concentration of total RNA. The 260/280 ratio > 1.8 and ≤ 2 were considered acceptable measures of RNA purity. cDNA was synthesized according to the manufacturer's instruction (Parstous Co., Iran) and was kept at −20°C for the next step. A Magnetic Induction Cycler (Mic) PCR Machine (micPCR) and SYBR Premix Ex Taq II (Ampliqon, Denmark) were used to evaluate the expression of target genes, Table 1. Primers for target genes were designed using Gene runner software version 6.5.52 (http://www.generunner.net/). The RT‐qPCR reaction was performed for each transcript according to the time cycle of the kit, which included a cycle of 95°C for 15 min, followed by 40 cycles at 95°C, 60°C, and 72°C for 20, 30, and 30 s. Finally, the expression of the target genes was measured using the 2^−ΔΔCt^ method. Glyceraldehyde‐3‐phosphate dehydrogenase (*Gapdh*) was a reference gene [23].

**TABLE 1:** Primer sequence of candidate genes.

Gene	NCBI reference Wsequence	Primer sequence (5′‐3′)	Product size (bp)
*Gapdh*	NM_017008.4	Forward: AAGGTCGGTGTGAACGGA	155
		Reverse: CTGAGATGGGTGCCGTTCAAG	
*Nrf2*	NM_001399173.1	Forward: TCTTCCATTTACGGAGACC	89
		Reverse: CCTTCACAGTTTGTCTTACC	
*HO‐1*	NM_012580.2	Forward: GAGATAGAGCGAAACAAGC	139
		Reverse: GACCACACATTCCCTACC	

Abbreviations: *Gapdh*, glyceraldehydes‐3‐phosphate dehydrogenase, *Nrf2*, nuclear factor (erythroid‐derived 2)‐like 2 *(Nrf2)*; *HO‐1*, heme oxygenase 1.

## 3 Statistical Analysis

GraphPad Prism (version 8.4.3) was used for data analysis and graph drawing. All behavioral and molecular test results are reported as mean ± SD. After evaluating the normal distribution of the data, a one‐way ANOVA analysis of variance followed by Tukey's *post hoc* test was used. The significance level for all groups was set at *p* ≤ 0.05.

## 4 Results

### 4.1 The Effects of Fenoprofen on Seizure Parameters

**FIGURE 2:**
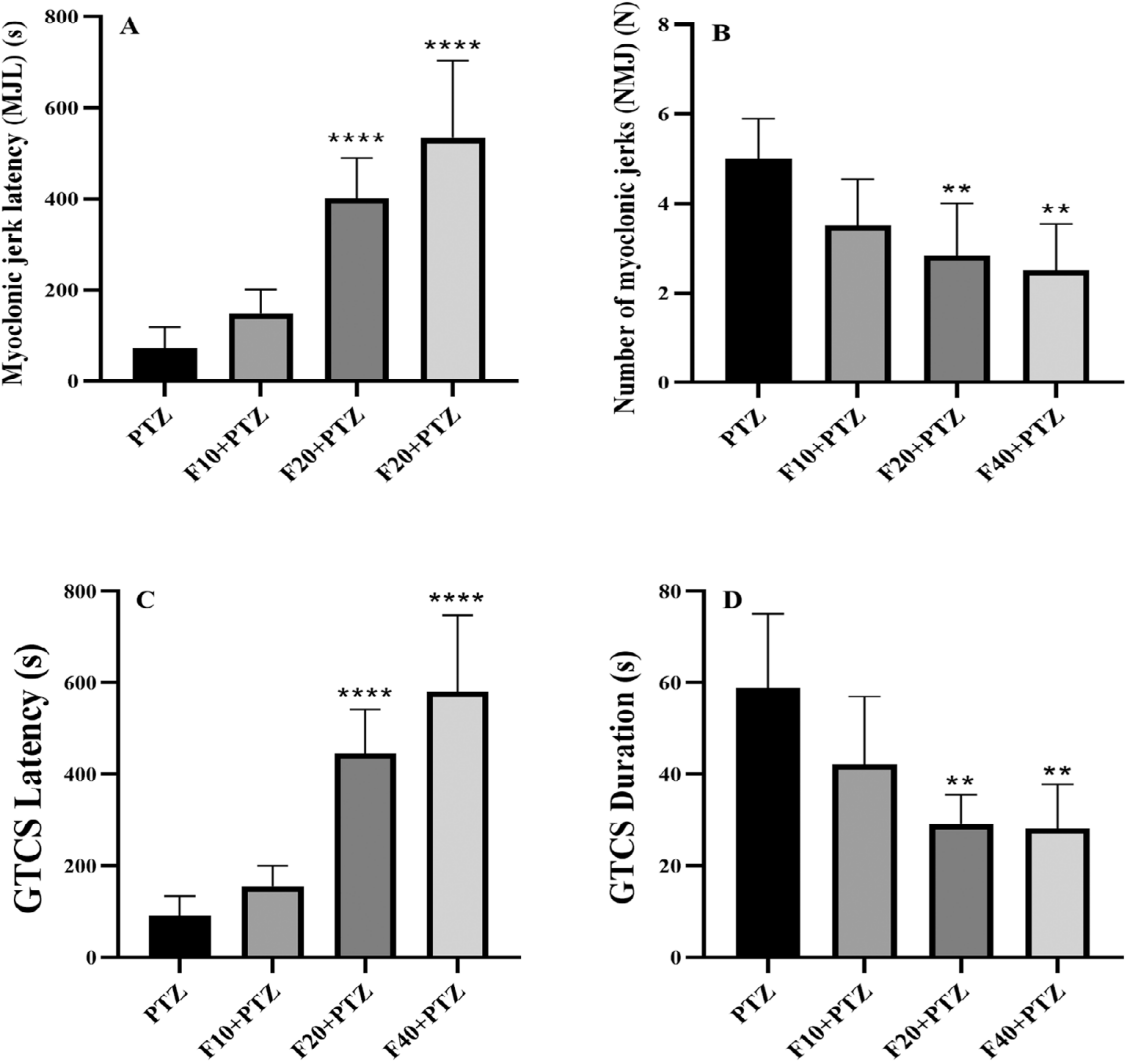
Effect of fenoprofen (10, 20, and 40 mg/kg) pretreatment on convulsive behaviors induced by PTZ injection. The MJL and NMJ are shown in Figure A and B, respectively. Figure C and D also show the delay and duration of GTCSs in experimental groups. ** indicates the comparison with the PTZ group *p* ≤ 0.01 and ****: *p* ≤ 0.0001. All results are reported as mean ± SD.

The results of fenoprofen treatment on convulsive behaviors induced by PTZ are shown in Figure 2A–D. Based on the analysis, there was a significant increase in MJL in the Fen20 and Fen40 groups compared to the PTZ group (*p* < 0.0001), [*F* (3,20) = 26.821, *p* < 0.0001], (Figure 2A). In the mentioned groups, NMJ also decreased significantly compared with the PTZ group, (*p* = 0.0091 and *p* = 0.0026, respectively), (*F* [3,20] = 6.756, *p* = 0.0025), (Figure 2B). GTCS‐L in Fen20 and Fen40 groups increased significantly (*p* < 0.0001), (*F* [3,20] =31.70, *p* < 0.0001). At the same time, GTCS‐D had a significant decrease compared with the PTZ group (*p* = 0.0026 and *p* = 0.0019, respectively), [*F* (3,20) =7.987, *p* = 0.0011], (Figure 2C,D).

### 4.2 The Effects of Fenoprofen Treatment on Oxidative Stress Markers

**FIGURE 3:**
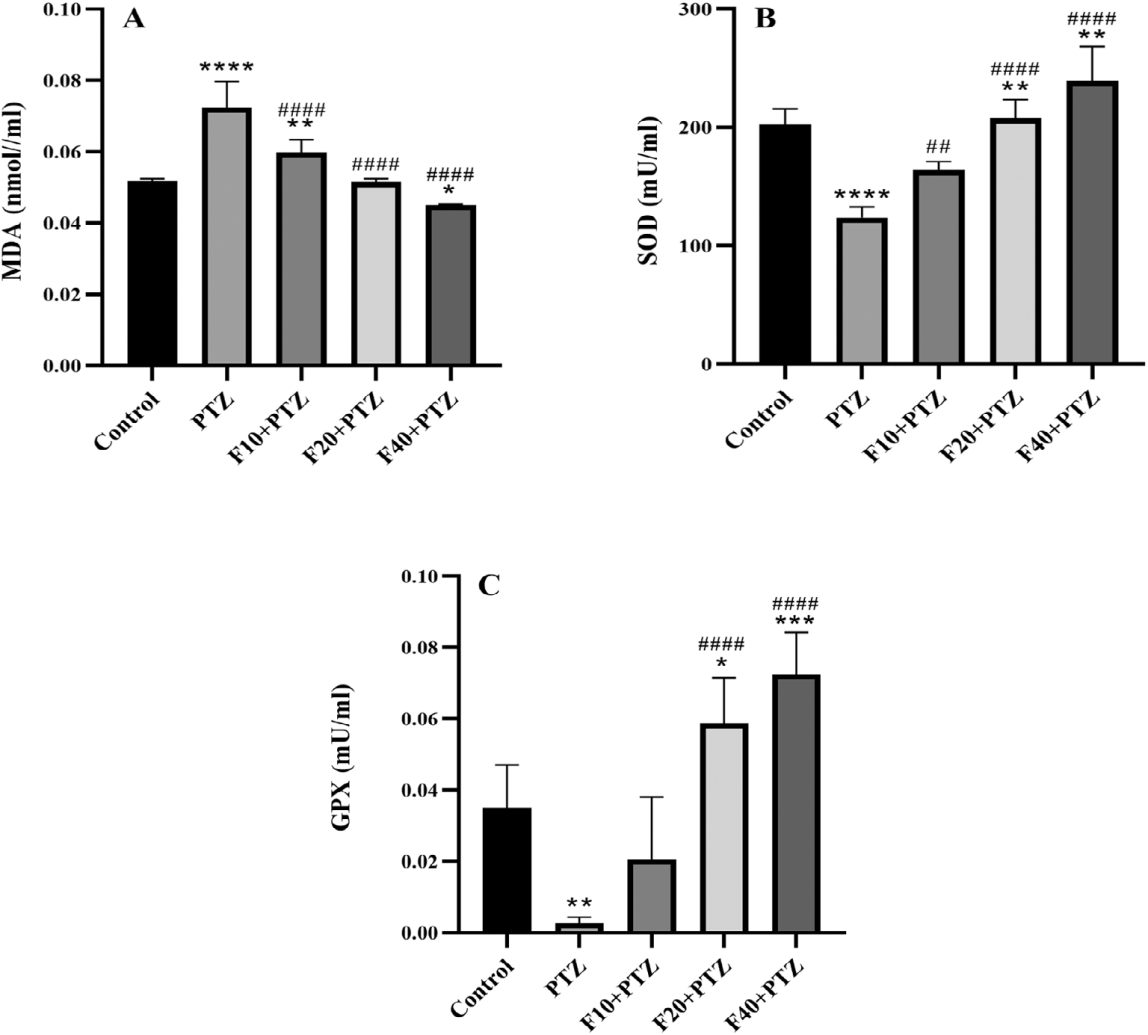
Effect of injecting different doses of fenoprofen on OS markers in the serum samples of epileptic rats. The levels of MDA, SOD, and GPX are shown in A, B, and C, respectively. * indicates the comparison with the control group *p* ≤ 0.05, **p ≤ 0.01, ***p ≤ 0.001, and ****p ≤ 0.0001. ^##^shows the comparison of groups with PTZ group *p* ≤ 0.01, ^###^ *p* ≤ 0.001. All results are reported as mean ± SD.

Data analysis revealed that there was a considerable difference in MDA levels among experimental groups (*F* [4,25] = 50.05, *p* < 0.000). Post hoc test revealed a significant increase in serum MDA levels in PTZ and Fen10 groups than the control group (*p* < 0.0001 and *p* = 0.0066, respectively), but in the Fen40 group, MDA level decreased significantly (*p* = 0.029). Furthermore, The MDA level in all experimental groups decreased considerably compared to the PTZ group (*p* < 0.0001), Figure 3A.

Compared with the control group SOD in the Fen40 group increased significantly (*p* = 0.0063) but in PTZ and Fen10 groups, it showed a significant decrease (*p* < 0.0001 and *p* = 0.0042, respectively). In serum samples of all fenoprofen‐treated groups, SOD increased significantly compared with the PTZ group (*p* = 0.0026, *p* < 0.0001, and *p* < 0.0001, respectively), (*F* [4,25] = 42.77, *p* < 0.0001), (Figure 3B). The results also showed a considerable decrease in GPX level in the PTZ group compared with the control group (*p* = 0.001). However, it increased significantly in the Fen20 and Fen40 groups (*p* = 0.020 and *p* = 0.0002, respectively). Also, in comparison with the PTZ group, GPX level increased in the Fen20 and Fen40 groups (*p* < 0.0001), (*F* [4,25] = 31.57, *p* < 0.0001), (Figure 3C).

### 4.3 Effect of Fenoprofen Treatment on the Morphological Change in the CA1 and CA3 Regions of the Hippocampus

**FIGURE 4:**
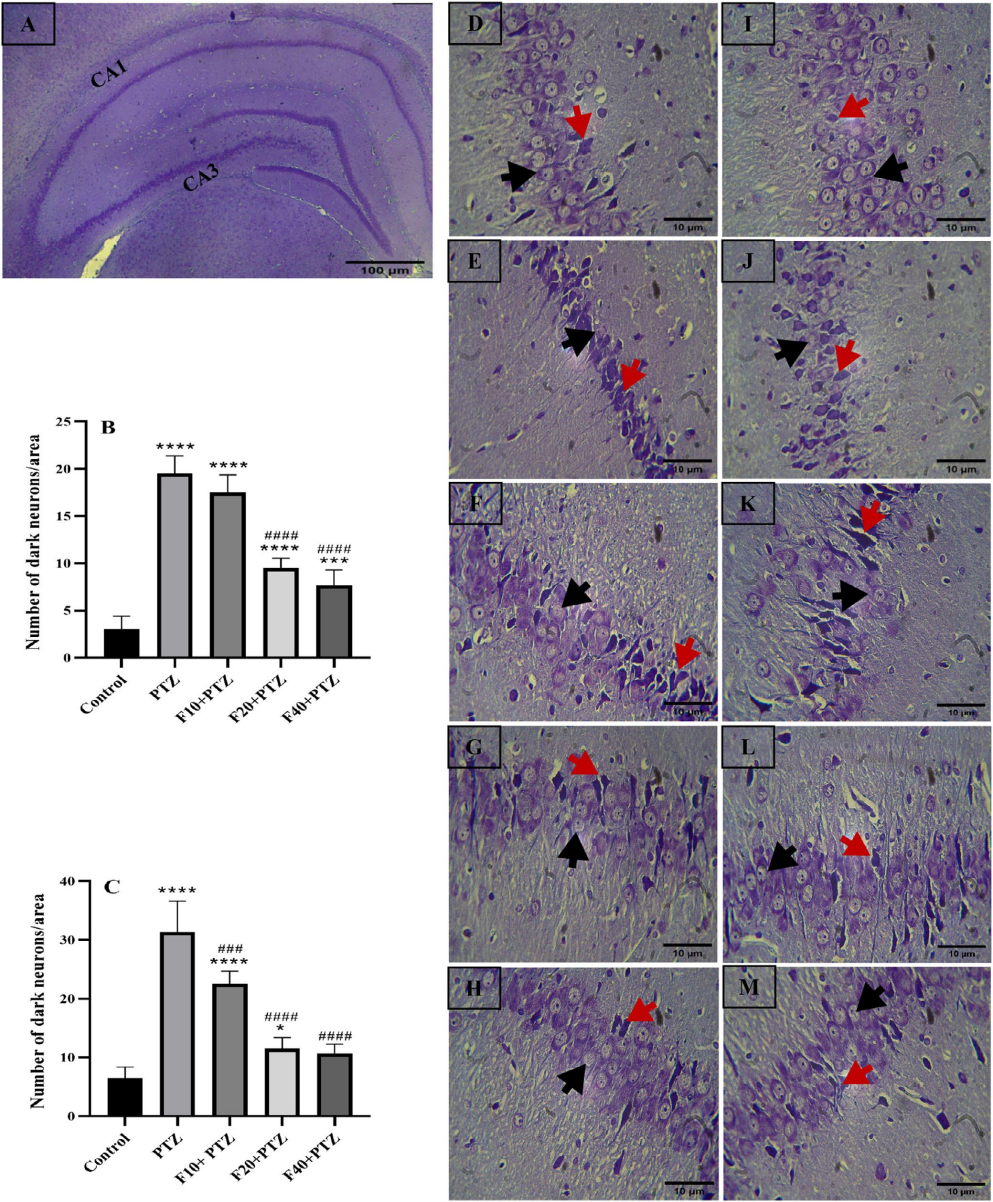
(A) Results of Nissl staining in the hippocampus with 10x magnification, and the CA1 and CA3 regions are shown. The number of dark neurons in the CA1 and CA3 regions were counted after recording the image with 40× magnification and the results are shown in (B) (CA1 region) and (C) (CA3 region). The * indicates the comparison with the control group, *p* ≤ 0.5, ***p ≤ 0.001, ****p ≤ 0.0001. ^####^represents the comparison with the PTZ control group, *p* ≤ 0.0001. The results are reported as mean ± SD. (D–H) shows the results of Nissl staining in the CA1 and (I–M) in the CA3 region. (D) Control group, (E) PTZ group, (F) Fenoprofen 10 (mg/kg) + PTZ group, (G) Fenoprofen 20 (mg/kg) + PTZ group, (H) Fenoprofen 40 (mg/kg) + PTZ group, (I) Control group, (J) PTZ group, (K) Fenoprofen 10 (mg/kg) + PTZ group, (L) Fenoprofen 20 (mg/kg) + PTZ group, (M) Fenoprofen 40 (mg/kg) + PTZ group. The black arrows indicate normal neurons and the red arrows indicate dark neurons.

Morphological changes in the structure of brain neurons are among the most critical consequences of excessive excitability. Compressed cell bodies, karyoplasms like necrotic cells, and dense cytoplasm are the characteristics of dark neurons. They are created under pathological conditions, including epilepsy and hippocampal sclerosis, and it seems that excessive secretion of neurotransmitters causes the formation of these neurons. These changes cause neurological dysfunction and poor cognitive function [24]. Nissl staining results of the coronal section of the CA1 and CA3 regions are shown in Figure 4A, with 10× magnification, and Figure 4D–M, with 40× magnification. As shown in Figure 4B, the number of dark neurons in the CA1 region of all experimental groups increased significantly compared with the control group (*p* < 0.0001, *p* < 0.0001, *p* < 0.0001, and *p* = 0.0003, respectively). However, compared with the PTZ group, these neurons in the Fen 20 and Fen 40 groups decreased considerably (*p* < 0.0001), [*F* (4, 25) =112.2, *p* < 0.0001]. Figure 4D–H shows the CA1 region. In the control group (Figure 4D), neurons are densely and coherently placed next to each other, the accumulation of Nissl bodies in the cytoplasm is well seen, and the nuclei are stained in blue. Also, a limited number of dark neurons can be seen in this area. Moreover, in the PTZ and Fen10 groups, the arrangement of neurons was irregular, neuronal coherence was lost, and the Nissl bodies were not visible (red arrows) (Figure 4E,F). In the Fen20 and Fen40 groups, better neuronal coherence is seen. In addition, Nissl bodies are well arranged (black arrows) (Figure 4G,H).

In the CA3 region, compared with the control group, the number of dark neurons in PTZ, Fen 10, and Fen20 groups increased significantly (*p* < 0.0001, *p* < 0.0001, and *p* = 0.0293 respectively). However, the number of these neurons in all fenoprofen‐treated groups showed a considerable decrease compared with the PTZ group (*p* = 0.0001, *p* < 0.0001, and *p* < 0.0001, respectively), [*F* (4,25) = 87.65, *p* < 0.0001], (Figure 4C). As shown in Figure 4I, the accumulation of Nissl bodies in the cytoplasm is well seen, the cell bodies are normal and the neuronal organization is preserved. However, in the PTZ and Fen10 groups, with a considerable increase in the number of dark neurons, the accumulation of Nissl bodies is not well seen, and the organization of neurons is lost (Figure 4J,K). In the F20 and F40 groups, the Nissl bodies in the cytoplasm are more visible than the PTZ group (Figure 4L,M). Moreover, most cell bodies are normal, and the neurons are more coherent.

### 4.4 Effect of Fenoprofen on *Nrf2*/*HO‐1* Gene Expression

**FIGURE 5:**
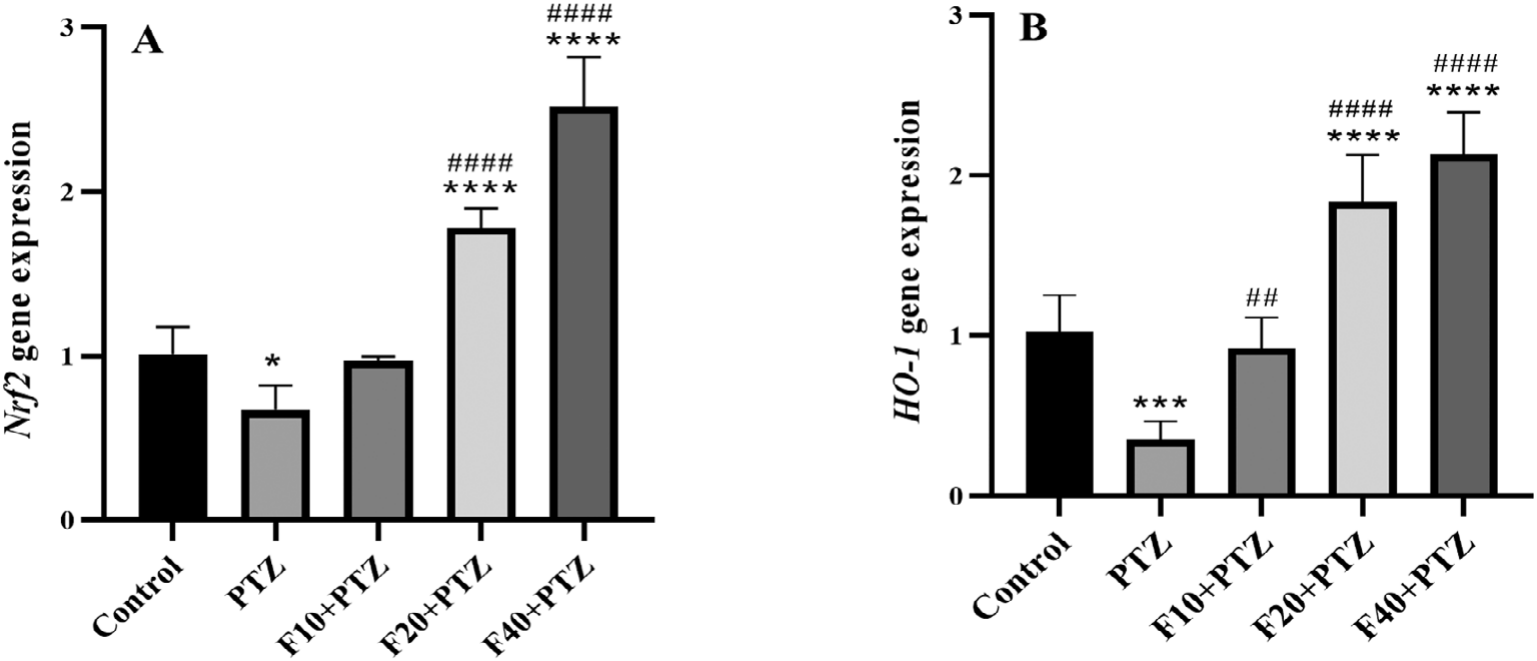
Effect of injecting different doses of fenoprofen on Nrf2 (A) and Ho‐1 (B) gene expression in the hippocampus of the experimental groups. * indicates the comparison with the control group *p* ≤ 0.05, ***p ≤ 0.001, and ^****^ *p* ≤ 0.0001. ^##^shows the comparison of the groups with PTZ group *p* ≤ 0.01, and ^####^ *p* ≤ 0.0001. All results are reported as mean ± SD.

As indicated in Figure 5A, *Nrf2* gene expression in the PTZ group, considerably decreased compared with the control group, (*p* < 0.0195), however, in the Fen20 and Fen40 groups, its expression was increased significantly (*p* < 0.0001). Compared with the PTZ groups, *Nrf2* gene expression in the Fen20 and Fen40 groups showed a considerable increase (*p* < 0.0001), (*F* [4,25] = 110.389, *p* < 0.0001). The results also showed a reduction in the relative expression of the *HO*‐*1* gene in the PTZ group compared with the control group (*p* = 0.0002). However, it increased significantly in the Fen20 and Fen40 groups (*p* < 0.0001). In addition, compared with the PTZ group, all experimental groups showed increased expression of the *HO‐1* gene (*p* = 0.0018, *p* < 0.0001, and *p* < 0.0001, respectively) [*F* (4,25) = 60.174, *p* < 0.0001], Figure 5B.

## 5 Discussion

Based on the results, fenoprofen pretreatment significantly protected against the PTZ‐induced kindling model of epilepsy and reduced the severity of convulsive behaviors. In treated groups, NMJ was decreased and the latency of myoclonic jerks onset was prolonged. It also increased GTCS latency and decreased its duration. These results show a reduction in the severity of convulsive behaviors in the pretreated groups. Previous evidence revealed that generalized convulsions and OS caused by PTZ alter the antioxidant defense system, induce cognitive impairments, provoke neurodegeneration, and activate redox‐regulated transcription factors. Such reactions stimulate the brain microenvironment for seizure activities [4, 25]. Although there is no specific study on the anticonvulsant effects of fenoprofen, the anticonvulsant effects of various NSAIDs, such as Oxaprozin [26] and Celecoxib [27, 28], in inducing neuroprotection, reducing seizure scores, improving cognitive behaviors, and decreasing OS markers have been well demonstrated in the animal model of epilepsy. These findings showed that NSAIDs by employing such mechanisms decline seizure severity and duration in animal models of epilepsy. In addition, there is evidence that treatment with Nimesulide, an NSAID, significantly diminished the PTZ‐induced kindling score. The authors stated that these effects were due to a decrease in the level of OS markers and an increase in antioxidant activity [29]. It should be noted that some NSAIDs such as Rofecoxib have shown a dose‐dependent effect on the severity of seizure activities. Low doses of Rofecoxib did not change the severity of seizures or the seizure threshold following intraperitoneal injection of PTZ. However, high doses of Rofecoxib caused a greater delay in the onset of convulsive seizures and reduced their severity [30].

Several mechanisms have been reported on how epilepsy is induced following PTZ injection. One of these mechanisms is a significant reduction in GABAergic neurons and an increase in the activity of the Glutamatergic system, which causes hyperexcitability. However, treatment with NSAIDs has been shown to upregulate GABAa receptors, enhance inhibitory currents, and inhibit the MAPK/ERK signaling pathway [31]. A bidirectional connection exists between NMDA glutamate receptors and MAPK/ERK signaling, which strengthens excitatory currents and hyperexcitability. In addition to affecting such signaling pathways, NSAIDs also potentiate inhibitory currents by acting on potassium channels [32, 33] and reduce calcium currents by inhibiting PG receptors. Thus these drugs prevent the release of excitatory neurotransmitters and inhibit hyperexcitability [34]. Therefore, part of the effects of fenoprofen in reducing the severity of convulsive behaviors like other NSAIDs seems to be through these mechanisms.

It is well‐accepted that, the induction of epilepsy increases free radicals in the brain and serum samples of laboratory models. Free radicals disrupt intracellular calcium homeostasis and affect synaptic transmission and neuronal excitability. They also make neurons more vulnerable by diminishing the energy level and inducing neuronal cell death [21, 35]. Based on the previous evidence, the increase of MDA, like other OS markers, exerts its effects by damaging biological membranes, inducing neurodegeneration, and disrupting cell signaling pathways [36]. MDA as the final product of lipid peroxidation, with its strong oxidative effects also exerts cytotoxic and mutagenic properties and changes cell [37]. In the current study, the increase in MDA level following PTZ injection was reduced after fenoprofen pretreatment. In treated groups, a significant decrease in the serum level of MDA was seen, which seems to be involved in the induction of the anticonvulsant effects of fenoprofen. Similar to our results other NSAIDs such as Ibuprofen were shown to suppress MDA production and exert antioxidant activity [38]. Moreover, another study revealed that Ibuprofen reduces the expression and activity of cyclooxygenase 2 and, by inhibiting the activation of NOD‐like receptor 3 inflammasome and IL‐18 secretion, reduces neuronal excitability, and seizure frequency and severity. It also minimized the loss of neurons in the hippocampus, indicating its protective effects [39].

Besides, SOD and GPx, which are immune system antioxidants, also prevent OS with their electron‐scavenging properties. Superoxide, or oxygen radicals, are converted into H_2_O and O_2_ by SOD activity. SOD contains various metal ions that can change the state of its electron transfer capacity. For example, the Fenton reaction converts the increased level of H_2_O_2_ in the presence of Fe^2+^ into hydroxyl radicals. Catalase in peroxisomes and GPx in mitochondria convert H_2_O_2_ into molecular oxygen and H_2_O and detoxify free radicals. Therefore, the antioxidant defense systems utilize different mechanisms and cause cell protection against free radicals [40]. So, it seems that strengthening the antioxidant defense system, which also reduces inflammation, can be considered a strategy to minimize convulsive behavior. According to the previous findings, the present study also showed that the levels of SOD and GPx in the serum samples of fenoprofen‐pretreated groups not only returned to normal state but also increased to some extent compared with the control group. Also, the considerable increase of MDA in the PTZ group was significantly reduced following fenoprofen pretreatment.

In epileptic seizure, brain areas also undergo morphological changes, including neuronal reduction, deformation, elevation of dark neurons, neuronal shrinkage, pyknosis, reduction in Nissl bodies, and disruption of neuronal organization. These changes, in turn, reduce long‐term potentiation and induce cognitive impairments. It is well‐accepted that the CA1 and CA3 regions of the hippocampus are critical regions targeted by epileptic seizures [41]. In a pilocarpine‐induced epilepsy model, posttreatment with celecoxib in addition to reducing the duration and frequency of seizures prevented neuronal damage in hippocampal regions, inhibited gliogenesis in the CA1 area, and reduced the number of active neuroglial cells in the hilus and CA1 regions [42]. In another epileptic model, it was revealed that treatment with Parecoxib, an NSAID, inhibits the production of inflammatory mediators and reduces neuronal damage in the piriform cortex and hippocampus [43]. In line with previous studies, our histological results obtained from Nissl staining showed that PTZ destroyed the neuronal organization of the CA1 and CA3 regions and increased the number of dark neurons. In addition to the loss of neuronal integrity, the number of wrinkled neurons in the PTZ group increased significantly, and the accumulation of Nissl bodies considerably decreased. However, dark neurons decreased in fenoprofen‐pretreated groups, and neuronal integrity was somewhat preserved. In addition, the accumulation of Nissl bodies in the cytoplasm of pretreated groups was almost similar to the control group, and the nuclei of the cells were well‐defined; however, detecting Nissl bodies in the PTZ group was impossible, especially in dark neurons. The neuroprotective effect of fenoprofen on hippocampal neurons may be involved in the anticonvulsant effects of fenoprofen.

Nrf2/HO‐1 signaling pathway were also examined in the hippocampus. In response to OS, many genes regulate free radical levels, intracellular redox homeostasis, and maintain mitochondrial integrity [44]. As one of the main regulators of the intracellular antioxidant defense system, Nrf2 is activated following electrophilic stress. It is then translocated to the nucleus and binds to the antioxidant response element promoters of various antioxidant genes, such as *HO*‐*1*. The Nrf2/HO‐1 signaling is a critical antioxidative pathway among cells. Moreover, several studies have shown that increased expression of *Nrf2/HO*‐*1* exerts protective effects against OS damage [45, 46]. Experimental models of various neurological diseases such as chronic ischemic (Khan, [47]), stroke [48], and Alzheimer's disease [49] have shown that inflammation and OS induce downregulation of the Nrf2/HO‐1 signaling pathway. Therefore, activation of Nrf2/HO‐1 signaling is an effective mechanism for suppressing OS‐related inflammatory reactions. As mentioned in the previous sections, antioxidant defense systems play a significant role in neutralizing the effects of free radicals and maintaining redox homeostasis; however, Nrf2/HO‐1 signaling is also an essential member of these endogenous regulatory cascades [50].

In our previous study on an epilepsy model, we showed that fenoprofen exerts neuroprotective effects by reducing inflammatory mediators such as TNF‐α and NF‐κB in the hippocampus and improves cognitive behaviors including anxiety‐like behaviors, memory, and learning [51]. Considering the two‐way connection between inflammation and OS, it seems that reducing OS markers also decreases inflammatory reactions and is involved in diminishing epileptic activities. There is evidence showing the potent anti‐inflammatory effects of celecoxib in epileptic models which seems to be mainly through the reduction in TNF‐α, IL‐1β, and COX‐2 expression. This, in turn, led to partial improvement of the disease [52]. The ability of NSAIDs to reduce/inhibit the synthesis of prostaglandins and other proinflammatory mediators such as TNF‐α and IL‐1β also appears to be involved in their antioxidative and anti‐inflammatory effects. In a study on PTZ‐induced seizures, it was observed that Ibuprofen using such mechanisms reduces Racine scores, seizure severity, and duration. Electroencephalogram recordings also confirmed lower amplitude and a reduced percentage of seizure spikes [53]. Our findings confirmed the same results so that decreased expression of *Nrf2* and *HO‐1* genes following PTZ treatment, increased after treatment with high doses of fenoprofen. Perhaps part of the effects of fenoprofen like other NSAIDs in reducing the severity of seizure behaviors is due to the strengthening of this signaling pathway.

Finally, given the high percentage of patients with drug‐resistant epilepsy and based on the obtained results in this study, it is recommended that further studies be conducted on the antiepileptic effects of fenoprofen. Also, many antiepileptic drugs show severe side effects, however, in this study, acceptable neuroprotective effects were observed at high doses, at least in the hippocampus. Although there is also evidence that NSAIDs have side effects in the long term, these effects can be controlled in various ways, such as synergistic use with other antiepileptic drugs or dose adjustment. Also, fenoprofen showed a dose‐dependent effect in most tests, so in future studies, higher doses can be used to measure its therapeutic effects or possible side effects. Moreover, this study was conducted on male Wistar rats, these effects can be investigated in other animal models. As mentioned in the discussion section, the antiepileptic effects of different NSAIDs on epilepsy models have been previously investigated. It is suggested that future studies also investigate the synergistic effects of fenoprofen with other NSAIDs.

## 6 Conclusion

In this study, for the first time, the antioxidant effects of fenoprofen and its possible mechanisms of action were investigated in the rat model of epilepsy. Based on the findings, pretreatment with fenoprofen caused neuroprotection in CA1 and CA3 areas of the hippocampus and strengthened antioxidant defense systems and Nrf2/HO‐1 signaling pathway. Fenoprofen appears to exert its anticonvulsant effects by targeting such factors. However, this study had some limitations, such as the small number of samples and lack of investigation of inflammatory factors, which can be investigated in future studies.

## Nomenclature

Key protein targets and ligands in this article are hyperlinked to corresponding entries in http://www.guidetopharmacology.org, the common portal for data from the IUPHAR/BPS Guide to PHARMACOLOGY, and are permanently archived in the Concise Guide to PHARMACOLOGY 2019/20 [54].

## Author Contributions

**Maryam Rahimi‐Tesiye:** conceptualization, methodology, data analysis, data curation, software, investigation, writing and editing the original draft. **Hassan Rajabi‐Maham:** conceptualization, investigation, supervision, resource, reviewing and editing the manuscript. **Vahid Azizi:** conceptualization, resource, supervision, visualization, reviewing and editing the manuscript. **Abdolkarim Hosseini:** conceptualization, methodology, software, reviewing and editing the manuscript.

## Ethics Statement

The study was performed in accordance with ARRIVE guidelines and was also approved by the local ethics committee of Shahid Beheshti University (code no. IR.SBU.REC.1402.122).

## Conflicts of Interest

The authors delcare no conflicts of interest.

## Data Availability

The data used and/or analyzed during the current study are available from the corresponding author upon reasonable request.
